# Identification of novel therapeutic targets in the PI3K/AKT/mTOR pathway in hepatocellular carcinoma using targeted next generation sequencing

**DOI:** 10.18632/oncotarget.1687

**Published:** 2014-02-21

**Authors:** Filip Janku, Ahmed O. Kaseb, Apostolia M. Tsimberidou, Robert A. Wolff, Razelle Kurzrock

**Affiliations:** ^1^ Departments of Investigational Cancer Therapeutics (Phase I Clinical Trials Program) and; ^2^ Gastrointestinal Medical Oncology, The University of Texas MD Anderson Cancer Center, Houston, TX; ^3^ Moores Cancer Center, The University of California San Diego, La Jolla, CA

**Keywords:** hepatocellular carcinoma, PI3K, AKT, mTOR, next generation sequencing

## Abstract

Understanding genetic aberrations in cancer leads to discovery of new targets for cancer therapies. The genomic landscape of hepatocellular carcinoma (HCC) has not been fully described. Therefore, patients with refractory advanced/metastatic HCC referred for experimental therapies, who had adequate tumor tissue available, had targeted next generation sequencing (NGS) of their tumor samples using the Illumina HiSeq 2000 platform (Foundation One, Foundation Medicine, MA) and their treatment outcomes were analyzed. In total, NGS was obtained for 14 patients (median number of prior therapies, 1) with advanced/metastatic HCC. Of these 14 patients, 10 (71%) were men, 4 (29%) women, 6 (43%) had hepatitis B or C-related HCC. NGS revealed at least 1 molecular abnormality in 12 patients (range 0-8, median 2). Detected molecular aberrations led to putative activation of the PI3K/AKT/mTOR pathway (n=3 [*mTOR*, *PIK3CA*, *NF1*]), Wnt pathway (n=6 [*CTNNA1*, *CTNNB1*]), MAPK pathway (n=2 [*MAP2K1*, *NRAS*]), and aberrant DNA repair mechanisms, cell cycle control and apoptosis (n=18 *[ATM*, *ATR*, *BAP1*, *CCND1*, *CDKN2A*, *CDK4*, *FGF3*, *FGF4*, *FGF19*, *MCL1*, *MDM2*, *RB1*, *TP53*]). Of the 3 patients with molecular aberrations putatively activating the PI3K/AKT/mTOR pathway, 2 received therapies including a mTOR inhibitor and all demonstrated therapeutic benefit ranging from a partial response to minor shrinkage per RECIST (-30%, -15%; respectively). In conclusion, genomic alterations are common in advanced HCC. Refractory patients with alterations putatively activating the PI3K/AKT/mTOR pathway demonstrated early signals of clinical activity when treated with therapies targeting mTOR.

## INTRODUCTION

The discovery of mutated “cancer genes”, has provided key insights into the mechanisms of tumorigenesis, which has been useful for developing targeted cancer therapies. Most recent examples include ALK inhibitors in non-small cell lung cancer with an *ALK* rearrangement or BRAF inhibitors in melanoma with a *BRAF* mutation.[1, 2]

Yet, hepatocellular carcinoma (HCC) is one of the most common malignancies worldwide and the third leading cause of death after lung and gastric cancers, but lacks molecular targets or signatures.[3] HCC comprises several distinct entities defined by etiology, and often arises in the wake of cirrhosis and liver dysfunction. Most cases of HCC are associated with hepatitis B or hepatitis C, as well as metabolic derangements such as alcoholic liver disease and nonalcoholic steatohepatitis. It is plausible that each entity may have a different molecular genetic profile that determines biological behavior, prognosis and response to molecularly targeted therapy.[4] Currently, complete resection or liver transplant are the only therapeutic approaches offering long-term survival. Most patients (>85%) with HCC present with advanced disease and about 12 months of survival on standard therapies.[5, 6] HCC is a highly heterogeneous tumor for which molecular signatures or druggable targets remain to be identified. According to the COSMIC database, which includes data from cell lines and tumor samples, there have been 1,709 oncogenes with oncogenic mutations identified in HCC to date (accessed 2/12/2013).[7] Mutations are primarily prevalent in *TP53* (31%), *CTNNB1* (19%), *AXIN1* (16%), *NFE2L2* (14%), *ARID2* (13%), and *PIK3CA* (7%).[7] However, systematic efforts to delineate the molecular profile of HCC in a large group of tumor samples from patients are still underway (http://cancergenome.nih.gov/).[8] Here we report the results of targeted next generation sequencing (NGS) and treatment outcomes in a case series of 14 patients with advanced/metastatic HCC.

## RESULTS

## PATIENTS

A total of 14 patients with advanced metastatic HCC were analyzed with NGS, of whom 10 (71%) were men and 4 (29%) women. Their median age at diagnosis was 58 years (range, 27 to 79 years) and 10 (71.5%) patients were White, 3 (21.5%) African American, and 1 (7%) Asian. In addition, 3 (21.5%) patients had hepatitis C-related HCC, 1 (7%) hepatitis B-related HCC, 2 (14.5%) hepatitis B and C-related HCC and 8 (57%) had HCC without identified predisposing factors. Patients received a median of 1 (range, 1-4) therapies prior to being referred for experimental therapies to the Clinical Center for Targeted Therapy (CCTT).

### Molecular profiling and treatment outcomes

**Figure 1:**
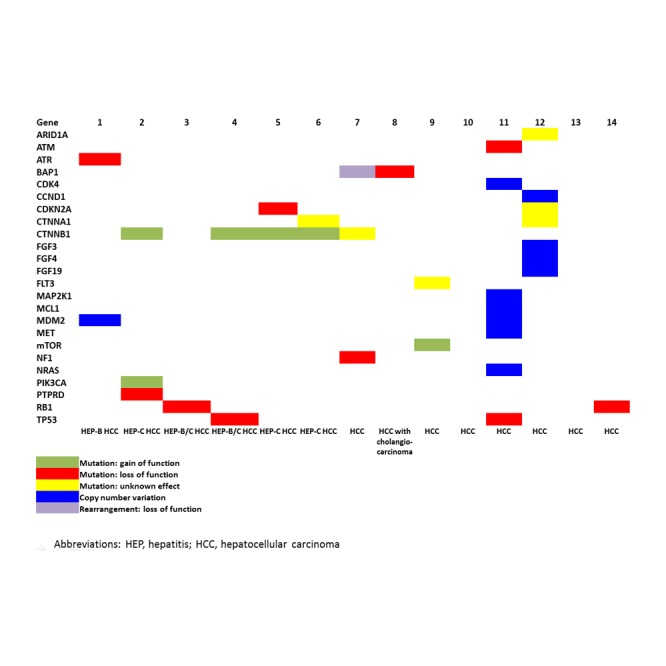
Overview of mutations in 14 patients with hepatocellular carcinoma The heatmap shows gain of function mutations (green), loss of function mutations (red), mutations with unknown effects (yellow), copy number variations (blue), and rearrangements (purple).

**Figure 2:**
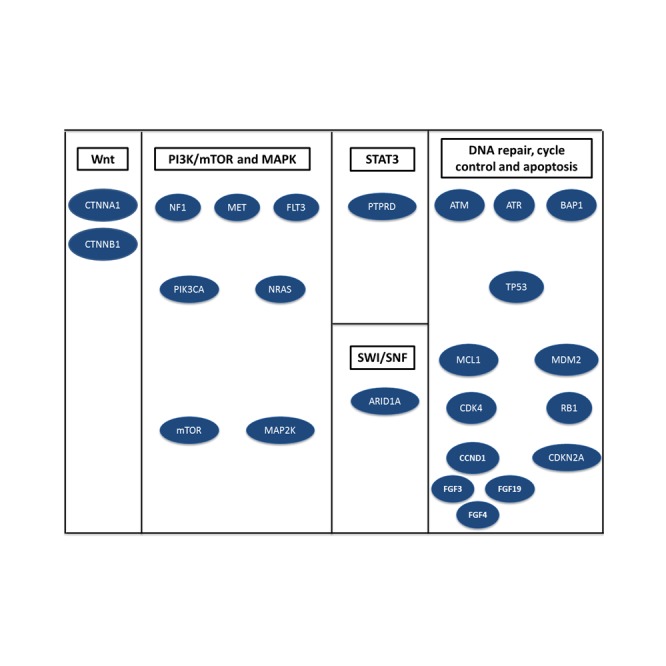
Major pathways altered by somatic mutations, copy number variations or rearrangements in 14 patients with hepatocellular carcinoma

Patient 1, an Asian male, was diagnosed at the age of 56 years with hepatitis B-related HCC and was initially treated with a liver transplant (Table 1). At the time of disease recurrence, the patient received firstline therapy with sorafenib and progressed after 4 months. NGS performed on a tumor sample from the epidural biopsy obtained after the firstline therapy revealed an *ATR* W463* mutation and *MDM2* amplification (Figure 1). *ATR* encodes the protein ataxia telangiectasia, which plays a key role in maintaining genomic integrity via regulation of DNA repair and replication (Figure 2).[9] The *ATR* W463*mutation truncates the *ATR* protein and is likely to lead to a loss of protein function. In HCC *ATR* mutations have been reported anecdotally.[7] Based on preclinical evidence, *ATR*-deficient tumors may be sensitive to PARP inhibitors.[9] *MDM2* acts to prevent the activity of the tumor suppressor p53; therefore, amplification of *MDM2* may be oncogenic (Figure 2). *MDM2* amplification has been identified according to some data in up to 44% of patients with HCC.[10] It is assumed that *MDM2* amplification may predict sensitivity to *MDM2* inhibitors; however, currently available evidence is inconclusive.[11] At the time of referral to the CCTT the patient had metastatic disease to lungs and bones and subsequently received experimental therapy with sorafenib (200 mg orally twice a day), temsirolimus (15 mg intravenously weekly), and bevacizumab (10 mg/kg intravenously every 3 weeks), which did not match any molecular target. Although the patient demonstrated a 25% improvement per RECIST after 8 weeks of therapy, he ultimately progressed in his bones after 2.8 months of therapy.

**Table 1:** Characteristics of patients with molecular aberrations who received experimental therapies

Patient number	Sex	Ethnicity	Age at diagnosis (years)	Subtype	Number of prior systemic therapies	Metastatic spread	Mutations (expected consequence)	Copy number variations	Rearrangements	Experimental therapy (target)	Therapy matching target	RECIST (%)	Time to progression (months)
1	Male	Asian	56	HCC, hepatitis B	1	Lungs, bones	ATR (impaired DNA repair and cell cycle control)	MDM2 (reduced apoptosis)	None	Sorafenib, temsirolimus, bevacizumab	No	−25	2.8
2	Male	African-American	51	HCC, hepatitis C, cirrhosis	1	Mediastinum, bones, liver	PIK3CA (PI3K/mTOR activation), CTNNB1 (Wnt activation), PTPRD (STAT3 activation)	None	None	Sirolimus (mTOR), vorinostat (HDAC)	Yes	−15	3.8
										Everolimus (mTOR), pazopanib (multikinase)	Yes	−11	2.9
										Erlotinib (EGFR), praletrexate (antifolate analog)	No	+11 (PD in non-target lesions)	2.1
6	Male	White	61	HCC, hepatitis C, cirrhosis	1	Liver, adrenal glands, bones	CTNNA1 (Wnt activation), CTNNB1 (Wnt activation)	None	None	Oxaliplatin (DNA damage), bevacizumab (VEGF), capecitabine (antimetabolite)	No	+13	3.2
7	Female	African-American	46	HCC	4	Lungs, mediastinum, liver, peritoneum	NF1 (PI3K/mTOR and MAPK activation), CTNNB1 (Wnt activation)	None	BAP1 (loss of interaction with BRCA1)	Everolimus (mTOR), pazopanib (multikinase)	Yes	−30	8.3
8	Female	African-American	56	HCC with cholangio-carcinoma	3	Lungs, mediastinum, liver, peritoneum, porta hepatis	BAP1 (loss of interaction with BRCA1)	None	None	Sirolimus (mTOR)	No	+130	1.4
										Nab-paclitaxel (mitotic inhibitor), bevacizumab (VEGF), gemcitabine (antimetabolite)	No	+15 (clinical PD)	1.6
12	Male	White	66	HCC	2	Liver	CDKN2A (loss of p16 function), ARID1A (SWI/SNF chromatin remodeling complex)	CCND1 (impaired cell cycle control), FGF3,4,19 (impaired cell cycle control)	None	MET kinase inhibitor	No	+14	4
14	Female	White	53	HCC	2	Lungs, mediastinum, liver	Rb1 loss (loss of cell cycle control)	None	None	Oxaliplatin (DNA damage), bevacizumab (VEGF), capecitabine (antimetabolite)	No	−18	2.9

Abbreviations: mTOR, mammalian target of rapamycin; HDAC, histone deacetylase; PI3K, phosphoinositide 3-kinase; EGFR, epidermal growth factor receptor

Patient 2, an African American male, was diagnosed at the age of 51 years with hepatitis C and cirrhosis-related HCC (Table 1). He progressed after 1.8 months of firstline therapy with sorafenib. NGS performed on a tumor sample from the chest wall biopsy obtained at the time of diagnosis revealed *PIK3CA* mutation H1047R, *CTNNB1* mutation S37C, and protein tyrosine phosphatase delta *(PTPRD)* mutation S1845fs*2 (Figure 1). *PIK3CA* encodes the active subunit of phosphatidylinositol 3-kinase (PI3K), which regulates cell growth, proliferation, differentiation, motility and survival (Figure 2).[12] Mutations in *PIK3CA* have been reported in 7% of liver cancer cases.[7] Preclinical and early clinical data suggest that activating mutations in *PIK3CA* may predict sensitivity to inhibitors of the PI3K/AKT mTOR pathway.[12-14] *CTNNB1* encodes beta-catenin, a key component of the Wnt signaling pathway (Figure 2). *CTNNB1* exon 3 mutations, such as S37C, are considered to be activating and lead to activation of the Wnt pathway.[15] *CTNNB1* mutations have been reported in 19% of HCC.[7] *PTPRD*, a tumor suppressor, plays an essential role in dephosphorylating STAT3 (Figure 2).[16] Mutations in *PTPRD* occur only sporadically in HCC (1%).[7] S1845fs*2 is a frameshift mutation that truncates the PTPRD protein within the second tyrosine phosphatase domain (amino acids 1644-1903) only 1 amino acid from the phosphatase active site, possibly resulting in an inactive protein. At the time of referral to the CCTT the patient had metastatic disease to the mediastinum, bones, and liver and subsequently received experimental therapy with a mTOR inhibitor sirolimus (4 mg orally daily) in combination with a histone deacetylase inhibitor (HDAC) vorinostat (300 mg orally daily), which was matching a molecular abnormality in the PI3K/AKT/mTOR pathway (*PIK3CA* H1047R mutation) and attained 15% shrinkage per RECIST. He progressed after 3.8 months.[17] Then he received a mTOR inhibitor everolimus (10 mg orally every other day) and multikinase inhibitor pazopanib (600 mg orally every other day), which again targeted the PI3K/AKT/mTOR pathway and induced 11% shrinkage per RECIST after 2 months, but he progressed after a total of 2.9 months. The patient then received the EGFR inhibitor erlotinib (100 mg orally daily) and anti*met*abolite praletrexate (15 mg/m^2^ intravenously weekly for 3 weeks followed by a week off), which did not match any of the detected abnormalities and progressed after 2.1 months with no initial response.

Patient 3, a White male, was diagnosed at the age of 59 years with hepatitis C-related HCC and history of hepatitis B. He was initially treated with a liver transplant. At the time of the second disease recurrence he progressed on firstline therapy with sorafenib after 5.9 months. NGS of a tumor sample obtained from a lung resection performed at the time of the first recurrence after the initial liver transplantation revealed *RB1* loss (Figure 1). *RB1* encodes the retinoblastoma protein, a tumor suppressor and negative regulator of the cell cycle (Figure 2).[18] The loss of *RB1* protein expression has been linked to poor survival and may prognosticate a decreased response to kinase inhibitors.[18] *RB1* mutations have been reported sporadically in HCC.[7] At the time of referral to the CCTT the patient had *met*astatic disease to the right pterygoid fossa and liver and his clinical condition precluded experimental therapy.

Patient 4, a White male, was diagnosed at the age of 57 years with hepatitis C, hepatitis B, and alcohol-related HCC. He was initially treated with liver transplantation and at the time of disease recurrence he received firstline therapy with sorafenib and progressed after 7 months. NGS performed on the tumor sample from the liver transplant after diagnosis revealed *CTNNB1* mutation I35S, and *TP53* V143M mutation (Figure 1). The *CTNNB1* gene plays a role in the Wnt signaling pathway and is characterized in detail above and the I35S mutation is considered to be activating (Figure 2).[15] Functional loss of tumor suppressor p53, which is encoded by the *TP53* gene, is common in advanced cancers (Figure 2).[19] *TP53* V143M mutation is located within the DNA-binding domain of the p53 protein, which is thought to result in loss of function.[20] Mutations of *TP53* have been reported in 31% of cases of HCC.[7] A recent meta-analysis reported that HCC patients with *TP53* alterations experienced significantly shorter recurrence-free survival and overall survival.[19] There are no approved therapies to address *TP53* mutations; however, tumors with *TP53* mutations may be sensitive to Wee1 inhibitors, which are in clinical trials. At the time of referral to the CCTT the patient had metastatic disease to the lungs, liver, retroperitoneum, and bones. He did not receive experimental therapy because of insurance denial.

Patient 5, a White male, was diagnosed at the age of 66 years with hepatitis C-related HCC. He was initially treated with sorafenib and progressed after 15.5 months; however, during that period of time he also underwent transarterial chemoembolization of the liver. NGS of a tumor sample obtained from a liver biopsy performed at the time of diagnosis revealed *CDKN2A* mutation H83Y and *CTNNB1* mutation T41A (Figure 1). The *CDKN2A* gene encodes the tumor suppressor p16(Ink4a), which plays a vital role in cell cycle G1 checkpoint regulation and its deletion contributes to inactivation of the p16-*CDK4*/Cyclin/Rb pathway and loss of cell cycle control (Figure 2).[21] The *CDKN2A* H83Y mutation leads to a reduced ability to induce cell cycle arrest. *CTNNB1*, a key component of the Wnt signaling pathway, is characterized above (Figure 2).[15] *CTNNB1* exon 3 mutations, such as T41A, are considered to be activating the Wnt pathway.[22] At the time of referral to the CCTT the patient had metastatic disease to the liver and decided to pursue therapeutic options outside of MD Anderson.

Patient 6, a White male, was diagnosed at the age of 61 years with hepatitis C-related HCC (Table 1). He was initially treated with sorafenib and progressed after 4.5 months. NGS of a tumor sample obtained from a bone biopsy performed at the time of diagnosis revealed *CTNNA1* mutation K889* and *CTNNB1* mutation T41A (Figure 1). The *CTNNA1* gene plays a role in cell adhesion downstream in the Wnt signaling pathway (Figure 2).[15] The *CTNNA1* K889* mutation is expected to be inactivating; however, its functional consequencies are not fully understood. *CTNNB1*, a key component of the Wnt signaling pathway, is characterized above (Figure 2).[15] *CTNNB1* exon 3 mutations, such as T41A, are considered to be activating the Wnt pathway.[22] At the time of referral to the CCTT the patient had metastatic disease to his liver, adrenal glands and bones and subsequently received experimental therapy with hepatic arterial infusion oxaliplatin (140 mg/m^2^ every 3 weeks), intravenous bevacizumab (10 mg/kg every 3 weeks) and oral; capecitabine (1500 mg/m^2^ on days 1-4 every 3 weeks) and progressed after 2.2 months. The experimental therapies did not match any detected molecular abnormality.

Patient 7, an African American female, was diagnosed at the age of 46 years with HCC (Table 1). She progressed on firstline therapy with sorafenib after 2.1 months. Then she progressed after 1.3 months of experimental therapy with an oncolytic virus and ultimately responded very well to a combination of bevacizumab and erlotinib, which she received twice with a PFS of 16.8 months and 10.1 months, respectively. NGS performed on a tumor sample from a liver biopsy obtained at the time of diagnosis revealed *NF1* mutation R1241*, *BAP1* truncation (exon 12), and *CTNNB1* mutation N387K (Figure 1). *NF1* encodes neurofibromin 1, a GTPase-activating protein (GAP) that is a key negative regulator of the RAS and PI3K signaling pathway (Figure 2).[23] The *NF1* R1241* mutation leads to a nonsense codon, resulting in truncation of the *NF1* protein within the GAP-related domain, which is likely to negate tumor suppressing activity. *NF1* mutations have been reported in 10% of HCC samples and such tumors may thus be sensitive to PI3K/AKT/mTOR and MAPK inhibitors.[7, 24] *BAP1* rearrangement results in a truncation of the BAP1 protein (Figure 2). The resulting truncated protein product is believed to be nonfunctional.[25] *BAP1* rearrangements have not been previously reported in HCC.[7] While there are no therapies that directly target *BAP1* loss, HDAC inhibitors are being explored based on the results of a preclinical study.[26] *CTNNB1*, a key component of the Wnt signaling pathway, is characterized above (Figure 2).[15] N387K is a rare mutation, located in the region responsible for binding to APC.[27] N387K has not been characterized, but other mutations in this region were shown to abrogate APC binding, although beta catenin was still degraded in the presence of these mutations.[27] The functional effect of N387K is unknown. At the time of referral to the CCTT the patient had *met*astatic disease to her lungs, mediastinum, peritoneum and liver and subsequently received the experimental therapy with mTOR inhibitor everolimus (7.5 mg orally every other day) and multikinase inhibitor pazopanib (600 mg orally every other day), which was matched to a molecular abnormality activating the PI3K/AKT/mTOR pathway (*NF1* R1241*mutation). Restaging CT scans revealed a PR (-30% per RECIST), which was maintained for the total of 8.3 months. Then the patient received mTOR inhibitor sirolimus (5 mg orally daily) and HDAC inhibitor vorinostat (300 mg orally daily), which again targeted the PI3K/AKT/mTOR pathway and was due to have her first reassessment after 2 months at the time of analysis.

Patient 8, an African American female, was diagnosed at the age of 56 years years with HCC with islands of cholangiocarcinoma (Table 1). She underwent a left hepatectomy and at the time of disease recurrence was treated with firstline therapy sorafenib and progressed after 10.7 months. Then she received a combination of oxaliplatin and gemcitabine and progressed after 6.6 months, followed by treatment with 5-fluorouracil, leucovorin, irinotecan, and bevacizumab, but progressed after 1.6 months. NGS performed on a tumor sample from the liver resection obtained at the time of diagnosis revealed *BAP1* mutation L65* (Figure 1). A *BAP1* L65* mutation in this tumor results in a truncation of the *BAP1* protein near the amino terminus. Details about *BAP1* are outlined above (Figure 2) [25]. At the time of referral to the CCTT the patient had metastatic disease to her lungs, mediastinal and hilar nodes, porta hepatis, peritoneum, and liver and subsequently received experimental therapy with mTOR inhibitor sirolimus (4 mg orally daily). She progressed after 1.4 months and then received hepatic arterial infusion nab-paclitaxel (180 mg/m^2^ every 3 weeks) and intravenous bevacizumab (10 mg/kg every 3 weeks) and gemcitabine (800 mg/m^2^ on days 1 and 8 every 3 weeks) and progressed after 1.6 months. None of the experimental therapies was matched to any detected molecular abnormalities.

Patient 9, a White male, was diagnosed at the age of 65 years with HCC, which was initially treated with a right hepatectomy and radiofrequency ablation. At the time of disease recurrence he was treated on firstline therapy with cisplatin, interferon, adriamycin, and 5-fluorouracil and remained progression-free for 23.7 months. He was next treated with capecitabine and progressed after 1.8 months, then treated on a clinical trial with regorafenib for 49.3 months and then with sorafenib, but progressed after 1.7 months. NGS performed on a tumor sample from the liver resection obtained at the time of diagnosis revealed *mTOR* mutation S2215Y and *FLT3* mutation splice site 1418+2A>G (Figure 1). mTOR acts downstream of multiple pathways, including the PI3K/AKT/mTOR pathway (Figure 2). This mTOR S2215Y mutation is located in the kinase domain.[28] The S2215Y mutation has been reported to result in constitutive activation of mTOR complex 1 (mTORC1) but not *mTOR*C2 and to retain sensitivity to sirolimus.[29] Mutations in *mTOR* have not yet been reported in HCC.[7] *FLT3* encodes a receptor tyrosine kinase. Signaling through the *FLT3* pathway leads to phosphorylation of SHC1 and AKT1 and activation of mTOR, as well as RAS activation and phosphorylation of ERK1 and 2 (Figure 2).[30] The 1418+2A>G splice site mutation seen in this tumor disrupts the canonical GT splice-donor site of the 5' end of the intron separating exon 11 and exon 12; however, the resulting GC dinucleotide sequence has been reported as a non-canonical splice site in the human genome.[31] Mutation of the conserved 5' GT splice site often results in exclusion of the neighboring exon, but may also result in truncation of the encoded protein.[32] This alteration has not been previously reported and its consequences are difficult to predict. Among FDA approved therapies sorafenib is known to be a *FLT3* inhibitor and regorafenib also has weak anti-*FLT3* activity.[33] At the time of referral to the CCTT the patient had metastatic disease to the liver and spleen. The patient did not receive experimental therapy due to his worsening performance status.

Patient 10 (White female diagnosed with HCC at the age of 67 years) had no molecular abnormality revealed through NGS.

Patient 11, a White male, was diagnosed at the age of 70 years with HCC, which was initially treated with a right partial hepatectomy. At the time of disease recurrence he was treated on firstline therapy with sorafenib and was taken off therapy after 4.6 months because of squamous cell carcinomas related to sorafenib therapy. NGS performed on a tumor sample from the liver resection performed at the time of disease recurrence revealed *ATM* mutation K53fs*3 and a *TP53* A161S and M160I mutations. In addition, *MET*, *CDK4*, *MAP2K1*, *MCL1*, *MDM2*, *NRAS* amplifications were found (Figure 1). *ATM* encodes the protein ataxia telangiectasia mutated, which plays a key role in sensing double-strand DNA breaks and activating cellular checkpoint pathways, arresting the cell cycle when DNA damage is present (Figure 2).[34] *ATM* K53fs*3 mutation is predicted to be inactivating due to loss of most of the ATM protein, including the kinase domain. According to the COSMIC database *ATM* mutations were found in all 4 tested HCC samples.[7] Preclinical evidence suggested that ATM deficient tumors may be sensitive to PARP inhibitors.[35] Functional loss of *TP53* is common in aggressive advanced cancers and is described in detail above (Figure 2).[19] Both A161 and M160 are located within the DNA-binding domain of TP5. While the A161S mutation is predicted to be severe, the M160I mutation is predicted to be non-severe.[36] DNA-binding domain mutations are thought to result in loss of function.[20] *MET* encodes a receptor tyrosine kinase that is activated by the ligand HGF, and MET activation promotes angiogenesis, resistance to apoptosis, proliferation, and invasion of cancer cells (Figure 2).[37] *MET* amplification has been reported in 2% of HCC.[38] *MET* amplification may predict sensitivity to *MET* inhibitors, some of which are FDA approved (crizotinib, cabozantinib).[39] *CDK4* encodes cyclin-dependent kinase 4, which, along with functional homolog CDK6 and family member CDK2, regulates cell cycle G1 phase progression and the G1/S transition (Figure 2).[40] *CDK4* amplification has been reported in 2% of HCC samples.[38] There are currently no approved therapies that directly target *CDK4*; however a number of drugs are under investigation in clinical trials. *MAP2K1* (also known as MEK1) encodes the signaling protein mitogen-activated protein kinase kinase 1. MEK1 phosphorylates the ERK1/2 proteins in the RAS/RAF/MEK pathway (Figure 2).[41] *MAP2K1* amplification has been reported in 2% of HCC samples.[38] The relationship between MEK amplification and sensitivity to MEK inhibitors is unknown. *MCL1* encodes the protein MCL1, which is a member of the BCL2 family and can result in antiapoptotic activity (Figure 2).[42] *MCL1* amplification has been reported in 12% of HCC.[38] There are no FDA approved therapies to address *MCL1* copy number amplification at this time, but investigations focused on small molecule inhibitors of *MCL1* are underway. In addition, the multikinase inhibitor sorafenib has been shown to down-regulate MCL1 and thereby induce apoptosis in preclinical studies.[43] Also, preclinical studies of patient-derived tumor cells suggest that increased MCL1 levels may confer resistance to antitubulin therapies such as paclitaxel.[44] MDM2 acts to prevent the activity of the tumor suppressor p53 and its function is described in detail above.[10] *NRAS* encodes a member of the RAS family of small GTPases that mediate transduction of growth signals (Figure 2).[41] Activation of RAS signaling causes cell growth, differentiation, and survival by activating the RAF/MAPK/ERK, PI3K, and other pathways. *NRAS* amplification has not been reported in HCC.[38] There are no approved therapies to address cancers associated with *NRAS* amplification or activating mutations. *NRAS* putatively leads to activation of the RAF/MEK/ERK pathway. Therefore, *NRAS* activation might theoretically predict sensitivity to MEK inhibitors. At the time of referral to the CCTT the patient had *met*astatic disease to the liver, but decided not to receive experimental therapy.

Patient 12, a White male, was diagnosed at the age of 79 years with idiopathic cirrhosis-related HCC (Table 1). He progressed after 2.7 months of firstline therapy with sorafenib. Then he received experimental therapy with bevacizumab and erlotinib and progressed after 3.8 months. NGS performed on a tumor sample from the liver biopsy obtained at the time of diagnosis revealed *CDKN2A* mutation A102P, *ARID1A* mutation I1485fs*5, *CCND1* amplification, *FGF19* amplification, *FGF3* amplification and *FGF4* amplification (Figure 1). *CDKN2A* regulates the p16-*CDK4*/Cyclin/Rb pathway and is characterized above (Figure 2).[21] The *CDKN2A* A102P mutation has not been characterized, but it is likely to be inactivating.[45] Overall, *CDKN2A* mutations has been reported in 8% of HCC.[7] *ARID1A* encodes the AT-rich interactive domain-containing protein 1A, a member of the SWI/SNF chromatin remodeling complex. *ARID1A* is believed to function as a tumor suppressor and deletion of *ARID1A* can lead to tumor formation.[46] The I1485fs*5 mutation results in truncation of the protein and similar truncations have been predicted to be inactivating.[46] *ARID1A* mutations have been reported in HCC in the COSMIC database.[7] *CCND1* encodes Cyclin D1, which interacts with the cyclin-dependent kinases *CDK4* and Cdk6, resulting in inactivation of *RB1* and progression of the cell cycle. Amplification of *CCND1* may therefore lead to increased proliferation. *CCND1* amplification has been reported in 4% of HCC.[47] *FGF3* encodes fibroblast growth factors 19, an FGFR4 ligand involved in regulation of hepatic protein and glycogen *met*abolism.[48] *FGF3* plays a central role in development of the inner ear and *FGF3* germline mutations give rise to an autosomal recessive syndrome characterized by microdontia, deafness and complete lack of inner ear structures.[49] *FGF4* plays a central role in tooth development.[50] At the time of referral to the CCTT the patient had *met*astatic disease to the liver and subsequently received experimental therapy with an oral daily dose of an experimental MET kinase inhibitor, which did not match any detected molecular abnormality. He had stable disease (+14% per RECIST) and progressed after 4.0 months.

Patient 13 (White male diagnosed with HCC at the age of 27 years) had no molecular abnormality revealed through NGS.

Patient 14, a White female, was diagnosed at the age of 53 years with HCC (Table 1). She progressed after 6 months of firstline therapy with sorafenib. Then she received experimental therapy with bevacizumab and erlotinib and progressed after 1.8 months. NGS performed on a tumor sample from the liver biopsy obtained at the time of diagnosis revealed *RB1* loss (Figure 1). *RB1* encoding the Rb protein is characterized above (Figure 2).[18] At the time of referral to the CCTT the patient had metastatic disease to her lungs, mediastinum and liver and subsequently received experimental therapy with hepatic arterial infusion oxaliplatin (140 mg/m^2^ every 3 weeks), intravenous bevacizumab (10 mg/kg every 3 weeks) and oral capecitabine (1500 mg/m^2^ on days 1-4 every 3 weeks) and was taken off therapy after 2.9 months because of poor tolerance. The experimental therapies did not match any detected molecular abnormality.

## DISCUSSION

Targeted NGS analysis of tumor samples from 14 consecutive patients with advanced HCC refractory to standard therapies referred to the CCTT for experimental therapies with targeted agents revealed that 12 (86%) patients had at least one somatic molecular aberration (median, 2; range, 0-8) in their tumor samples (activating mutations, n=6; loss of function mutations, n=10; mutations with unknown effects, n=6; copy number variations, n=11; rearrangements, n=1) and 9 (64%) had more than one molecular aberration. Of interest, molecular aberrations were found in the PI3K/AKT/mTOR pathway (*mTOR*, n=1; *PIK3CA*, n=1; *NF1*, n=1), Wnt pathway (*CTNNA1*, n=1, *CTNNB1*, n=5), the MAPK pathway (*MAP2K1*, n=1; *NRAS*, n=1), DNA repair mechanisms, cell cycle control and apoptosis (*ATM*, n=1, *ATR*, n=1; *BAP1*, n=2, *CCND1*, n=1; *CDKN2A*, n=2; *CDK4*, n=1; *FGF3*, n=1; *FGF4*, n=1, *FGF19*, n=1; *MCL1*, n=1; *MDM2*, n=2; *RB1*, n=2; *TP53*, n=2). Six (43%) patients with HCC in the context of hepatitis B (n=1), hepatitis C (n=3) or both (n=2) had molecular abnormalities related to the PI3K/AKT/mTOR pathway (*PIK3CA*, n=1), Wnt pathway (*CTNNA1*, n=1; *CTNNB1*, n=4), STAT3 signaling (*PTPRD*, n=1), DNA repair mechanisms, cell cycle control and apoptosis (*ATR*, n=1; *CDKN2A*, n=1; *RB1*, n=1; *MDM2*, n=1; *TP53*, n=1). Despite the fact that several patients had aberrations in some genes such as *CTTNB1* and *CDKN2A,* the specific combination of aberrations and their subtypes were unique to each individual patient, important distinctions for selecting personalized cancer therapies.

Patients with hepatitis B or C-related HCC had a median of 2 aberrations (range, 1-3), which was similar to a median of 1.5 aberration in patients without hepatitis (range, 0-8; p = 0.74). To date, the molecular landscape of HCC has not been clearly defined and major initiatives such as The Cancer Genome Atlas are in progress. Only limited data on copy number variations are available, demonstrating that *MCL1*, and *BAP1* are the most frequent copy number abnormalities (12%, 4%, respectively).[38] According to the COSMIC database, the genes most frequently mutated include *TP53* (31%), *CTNNB1* (19%), *AXIN1* (16%), *NFE2L2* (14%), *ARID2* (13%), and *PIK3CA* (7%).[7] An integrated analysis of somatic mutations and focal copy number variations with whole exome sequencing of 24 HCC cases revealed recurrent mutations in *CTNBB1* (46%), *AXIN1* (21%), *TP53* (13%), *CDKN2A* (8%), and *ARID2* (8%).[51] *CTTNB1* was also the most frequently mutated gene in our study (5/14, 36%). Of interest *mTOR* mutation S2215Y has never been reported before in HCC.

Of the 14 patients in our study, 3 (21%) had genomic alterations putatively activating the PI3K/AKT/mTOR pathway and 2 such patients received therapies that included an mTOR inhibitor. Both patients had some therapeutic benefit ranging from a PR to minor shrinkage per RECIST (-30%, -15%; respectively), which was maintained for 3.8 months and 8.3 months. According to the COSMIC database, with the exception of *PIK3CA,* none of the mutations putatively leading to PI3K/AKT/mTOR activation are commonly found in HCC; however, preclinical models and early clinical data generally suggest that targeting the PI3K/AKT/mTOR pathway can be an effective strategy in subsets of patients with HCC.[52, 53] However, none of the studies investigated these therapies in HCC with PI3K/AKT/mTOR activation.[54, 55] Revisiting such studies with gating of HCC patient for alterations in the PI3K/AKT/mTOR pathway may yield better evidence of the efficacy of inhibitors of the PI3K/mTOR pathway in this difficult to treat disease.[56-58]

In conclusion, we have identified multiple molecular abnormalities in a small data set of patients with advanced HCC. Interestingly, a proportion of our patients had molecular aberrations putatively associated with PI3K/AKT/mTOR pathway activation and these patients attained some benefit (although not always durable) from targeting the PI3K/AKT/mTOR pathway. Despite several limitations such as small patient numbers, absence of randomization and control groups, our data suggest that comprehensive molecular profiling can increase our knowledge of HCC biology to an unprecedented level, which can be translated to patient care.

## PATIENTS AND METHODS

### Patients

We reviewed the electronic medical records of consecutive patients with advanced/metastatic HCC (excluding fibrolamellar type given its distinct entity and natural history) referred to the CCTT at MD Anderson Cancer Center. Data were collected from transcribed notes and radiology reports in the electronic medical record and other source documentation. Registering patients in the database, clinical, pathologic, laboratory and pathology assessment were performed at MD Anderson. The study and all treatments were conducted in accordance with the guidelines of the MD Anderson Institutional Review Board.

### Molecular analysis

Archival tumor samples obtained from standard diagnostic or therapeutic procedures were tested with Clinical Laboratory Improvement Amendment-certified targeted NGS (Foundation One, Foundation Medicine, MA) using the Illumina HiSeq 2000 platform. Genomic libraries were captured for 3,230 exons representing the complete coding sequence of 182 cancer-related genes plus 37 introns from 14 genes often rearranged in cancer and sequenced to an average median depth of 734X with 99% of bases covered >100X. On January 1, 2013, the platform was upgraded to cover 3,769 exons of 236 cancer-related genes and 47 introns from 19 genes to an average depth of 1000X.

### Treatment

Patients were enrolled in clinical trials according to the discretion of treating physicians and availability of clinical trials at the time of enrollment. Treatment continued until disease progression or unacceptable toxicity. Treatment was carried out according to the specific requisites in the treatment protocols selected.

Assessments, including history, physical examination, and laboratory evaluations, were performed as specified in each protocol, typically before the initiation of therapy, weekly during the first cycle, and then, at a minimum, at the beginning of each new treatment cycle. Response was assessed from computed tomography (CT) scans and/or magnetic resonance imaging (MRI) at baseline before treatment initiation and then every 2 cycles (6-8 weeks). All radiographs were read in the Department of Radiology at MD Anderson and reviewed in the Department of Investigational Cancer Therapeutics tumor measurement clinic. Responses were categorized per RECIST criteria and categorized as complete response (CR); partial response (PR), stable disease (SD) or progressive disease (PD).[17]

### Statistical analysis

Mann-Whitney test was used to assess the association between continuous variables. All tests were two-sided, and P values less than 0.05 were considered statistically significant. All statistical analyses were carried out using SPSS 19 computer software (SPSS Chicago, IL).
